# Modeling measurement error in tumor characterization studies

**DOI:** 10.1186/1471-2105-12-284

**Published:** 2011-07-13

**Authors:** Cyril Rakovski, Daniel J Weisenberger, Paul Marjoram, Peter W Laird, Kimberly D Siegmund

**Affiliations:** 1 Department of Preventive Medicine, USC Keck School of Medicine, Los Angeles, California 90089-9176, USA; 2 Departments of Surgery and Biochemistry and Molecular Biology, USC Keck School of Medicine, Los Angeles, California 90089-9176, USA; 3 USC Epigenome Center, University of Southern California Norris Comprehensive Cancer Center, Los Angeles, California 90089-9176, USA; 4 Chapman University, Orange, California 92866, USA

## Abstract

**Background:**

Etiologic studies of cancer increasingly use molecular features such as gene expression, DNA methylation and sequence mutation to subclassify the cancer type. In large population-based studies, the tumor tissues available for study are archival specimens that provide variable amounts of amplifiable DNA for molecular analysis. As molecular features measured from small amounts of tumor DNA are inherently noisy, we propose a novel approach to improve statistical efficiency when comparing groups of samples. We illustrate the phenomenon using the MethyLight technology, applying our proposed analysis to compare *MLH1 *DNA methylation levels in males and females studied in the Colon Cancer Family Registry.

**Results:**

We introduce two methods for computing empirical weights to model heteroscedasticity that is caused by sampling variable quantities of DNA for molecular analysis. In a simulation study, we show that using these weights in a linear regression model is more powerful for identifying differentially methylated loci than standard regression analysis. The increase in power depends on the underlying relationship between variation in outcome measure and input DNA quantity in the study samples.

**Conclusions:**

Tumor characteristics measured from small amounts of tumor DNA are inherently noisy. We propose a statistical analysis that accounts for the measurement error due to sampling variation of the molecular feature and show how it can improve the power to detect differential characteristics between patient groups.

## Background

The molecular heterogeneity of cancer is explored through a variety of technologies. With the decreasing costs of technology, in combination with the development of methods for the analysis of archival specimens, it is now feasible to conduct molecular analysis in large epidemiologic studies. Of primary interest are the population-based frequencies of different molecular features, and whether they vary by patient characteristics. However, an issue that arises with the analysis of archival specimens is the varying quantity and quality of DNA material that they can provide. Real-time PCR using TaqMan^®^-based fluorescence chemistry is one technology often used for sensitive detection for measuring a number of such molecular features. Understanding the impact that a low amount of amplifiable material has on the stability of the lab measurement is important to efficient statistical analysis.

In cancer studies of archival specimens, tumors are sectioned, allowing them to be subject to many different types of molecular studies. Consequently, DNA is extracted from only a portion of the tumor, and fragmented prior to lab analysis. Both the heterogeneity in the tumor cell population, as well as the uncertainty due to sampling, will influence the estimate of the outcome measure and its stability. The issue of tumor heterogeneity is well-appreciated among researchers; the epigenetic variation of the pure tumor cell population is obscured by the inclusion of infiltrating cells in the tissue sample. However, it is not the impact of mixture cell populations that we discuss. Instead we focus on the second factor, one which is present even for populations of a single cell type, variation due to low amounts of amplifiable input DNA, or equivalently, small sample size. The precision of each lab measurement, reflecting the uncertainty due to sampling DNA fragments, typically goes unreported. We illustrate the effect of this uncertainty on the variance of the outcome measure, and propose new methods that incorporate the sampling uncertainty into the statistical analysis.

The issue of sampling variation from the molecular analysis of archival tissues first came to our attention in a study of *MLH1 *methylation in the Colon Cancer Family Registry (C-CFR) [1]. In the C-CFR, *MLH1 *DNA methylation was measured from paraffin-embedded sections for over 1,200 colorectal tumors using the highly sensitive MethyLight technology [2]. MethyLight utilizes TaqMan-based, real-time PCR technology, which can provide an approximate measure of the amount of methylated DNA from a gene-specific MethyLight reaction and the amount of bisulfite-converted DNA using a control reaction, from the number of PCR cycles, or C(t) value. High quantities of DNA require fewer PCR cycles to reach a detection threshold above background and measure molecular features (low C(t)), whereas low quantities of DNA require more PCR cycles (high C(t)). In the C-CFR study we saw great variability in the *ALU *C(t) levels across tumors, indicating a wide variation in amount of DNA input available for molecular analysis. However, it is not standard to report the distribution of the C(t) level for the control reaction in molecular studies [3,4] and without this information, it is unclear whether samples negative for DNA methylation truly lack methylation, or whether they lack a sufficient amount of DNA to detect it.

**Figure 1:**
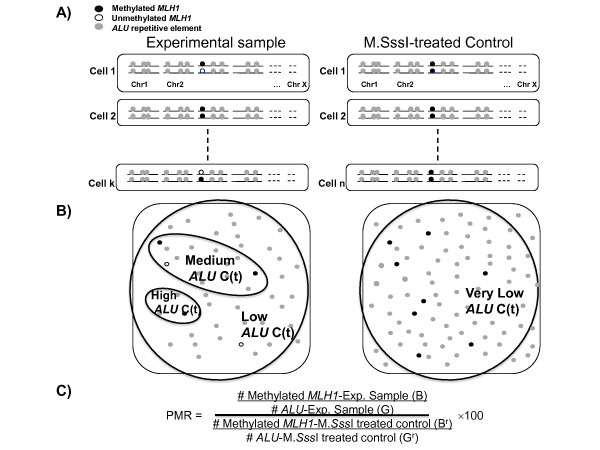
**Illustration of measuring DNA methylation using the MethyLight technology**. A) Example DNA from an experimental sample and an M.*Sss*I-treated control sample. In the M.*Sss*I-treated control sample all unmethylated MLH1 molecules become methylated. B) Sampling of DNA fragments. A large number of fragments sampled results in a very low real-time PCR cycle threshold (*ALU *C(t) value). A very small number of fragments sampled results in a high *ALU *C(t) value. C) The formula to compute Percent Methylated Reference (PMR), a ratio of methylated alleles in the experimental sample (black balls) over the quantity of input DNA (grey balls) in the experimental and reference samples.

Figure 1 shows a schema illustrating how DNA features are studied using MethyLight technology. A sample of DNA consists of a collection of DNA fragments, obtained from pooling the DNA from a number of cells. In our example, grey balls denote segments of DNA containing the sequence encoding *ALU *repetitive elements. These sequences occur frequently throughout the genome and help us quantify the amount of DNA present in each sample. The black and white balls represent DNA methylation states from the DNA target sequence of interest; black denotes fully methylated *MLH1 *promoter/5' gene region and white denotes otherwise. When DNA methylation is analyzed using MethyLight, measures from an experimental sample are compared to a reference, where the reference sample is treated with M.*Sss*I methylase, an enzyme which methylates all CpGs and therefore converts white balls to black balls. The outcome measurement is the percent of methylated reference (PMR), which can be viewed as the ratio of the methylated alleles in the tumor and reference samples adjusted (by scaling) for the different input DNA quantities and multiplied by 100 (Figure 1C, see Methods section for details). High PMR values denote high levels of concordant DNA methylation in the target sequence while PMR values of zero represent none detectable. When the quantity of input DNA from a tumor harbouring DNA methylation is inadequate, PMR values can have very large variance, erroneously resulting in PMR ratios of 0 (false-negatives) or ratios greater than 100. Variable levels of input DNA quantity are illustrated in Figure 1B.

By simulating the DNA sampling process, we illustrate the effect of quantity of amplifiable input DNA on the sampling variation in PMR value, and show that the variation of PMR increases with decreasing DNA quantity. For testing differential DNA methylation between groups, we propose a novel weighted regression approach, which uses as weights the inverse variance estimates of PMR as a function of a surrogate measure for DNA quantity. For the Colon CFR study of *MLH1 *methylation using MethyLight, we use the *ALU *C(t) value as the surrogate variable. Similarly, the use of A_260_nm-absorbance measurements may also provide an estimation of the amount of input DNA available in DNA methylation-based assays. Here, we present a simulation study to compare the newly proposed approach to a standard analysis that ignores measurement error due to sampling genomes. We then apply the different statistical approaches to real data from the Colon CFR.

## Methods

### PMR values using MethyLight

The PMR is defined as follows:

where *B *and *B*^*r *^are the numbers of methylated alleles for the tumor and reference samples, respectively, and *G *and *G*^*r *^are the corresponding DNA quantities. The letters B and G remind us of the black and grey balls described in Figure 1. The PMR value can be re-written as the ratio of the methylated alleles in the tumor and reference samples adjusted (by scaling) for the different DNA quantities (=*B/B*^*r *^× (*G*^*r*^/*G *× *100*)). A natural probabilistic model for the PMR variable is a scaled ratio of two binomial variates, where the scaling ratio is based on estimates of DNA quantity. Because the DNA is fragmented, the total number of genomes is not measured directly; instead, the amount of input DNA is quantified by studying interspersed repeat elements occurring throughout the genome, and scaling the genome to the number of measured repeats. We use the repetitive element *ALU *for this purpose [5].

**Figure 2:**
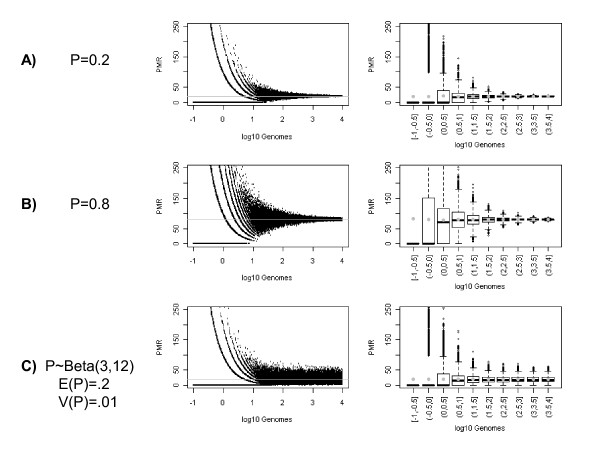
**Scatterplot of simulated data showing Percent Methylated Reference (PMR) against C(t) value for different true methylation proportions, p**. A) p = 0.2, B) p = 0.8, C) P ~ Beta(3,12). Grey lines in figures in the left column show the true methylation proportion (or its average), and grey dots in figures in the right column show the average PMR grouped by category of input DNA quantity.

We illustrate, through simulation, the variation in PMR as a function of DNA quantity for thousands of tumors (Figure 2). The data are simulated to mimic the process of sampling from the collection of DNA fragments in each sample. Let *h *denote the number of haploid genomes, and *p *the proportion of a genomic target that is methylated. The total number of DNA fragments in each sample is *G = h × f*, the product of the number of haploid genomes, *h*, times *f*, the number of DNA fragments per genome. Then, the number of methylated alleles, *B *for the tumor sample is simulated from a Binomial distribution with sample size *G*, and a methylation frequency of *p*/*f*, the proportion of DNA methylation at our genomic target scaled to the number of fragments per genome. In the M.*Sss*I-treated control sample, we assume that the target of interest is methylated in every genome (*p *= 1). Then the number of methylated alleles, *B*^*r*^, is simulated from a Binomial distribution with sample size *G*^*r*^, and DNA methylation frequency 1/*f*. We describe in the results section what happens if this assumption is not met and the DNA is not fully converted (p < 1). Previous research using MethyLight to measure the number of repetitive element *ALU *fragments, suggests that we can use *f *= 10^4 ^fragments per genome [5], giving us a DNA methylation frequency of *p*/10^4 ^in the tumor sample and 1/10^4 ^in the fully methylated control. Based on a supplementary study designed and conducted by our lab (see Additional file 1), we estimated that our reference samples contained 5,500 genomes. Using these as input, we simulated PMR values as follows:

where *h *is randomly sampled from a lognormal distribution, to mimic the distribution found in the Colon CFR study. The PMR is zero if and only if *B = 0*, which happens with probability Pr(*B = 0*) = (1-*p*)^*h*^. For *p *> 0, as the amount of input DNA approaches 0, Pr(*B = 0*) *→ 1*, causing an excess of PMRs = 0 and a high false-negative rate of detection.

### Differential DNA methylation analysis

We propose a novel weighted regression approach to study the association between exposure and PMR value, where the weights are based on an empirical estimate of the variance of PMR as a function of a surrogate measure for DNA quantity. This allows us a quantitative approach to handling measurement error from including samples with low amounts of DNA input in the analysis, beyond a simple inclusion/exclusion filter of the data. Two approaches to computing weights are presented. In the first, the data are divided into categories defined by quantiles of the surrogate variable for DNA quantity, and the variance in PMR is estimated for each category. In the second, we order the PMR measures by increasing value of the surrogate and compute the variance of the PMRs for sliding windows. We used different numbers of categories and window sizes in simulated data and found that for a sample size of 200, grouping the data by quintiles, with 40 observations in each category, gave good power to detect differential DNA methylation, while allowing us to control the false-positive rate. For the second approach, we chose a window size of 41 so that there were approximately the same numbers of observations per group as for our categorical approach. When possible, we centered the window around the observation of interest and computed the variance including the 20 observations ranked just above and the 20 ranked just below the observation of interest (ranking based on the surrogate variable for DNA quantity). For the 20 highest and lowest ranked observations for which we could not center the window, we computed variance using the 41 top-most, or bottom-most, observations, respectively. For each observation, the inverse variance for that quintile of DNA quantity, or window, provides the weights in a weighted least squares (WLS) regression. We refer to the two WLS approaches as WLS-Q and WLS-W, depending on whether the weights are estimated using quintiles of DNA quantity, or a window, respectively.

In a simulation study, the test size and power to detect differential DNA methylation between groups of tissues are compared. In order to present results on an absolute scale, we report results using cut-offs based on the simulated DNA quantity. This allows us to interpret the results in terms of the biologically relevant scale of genome equivalents, instead of indirectly through a surrogate variable. However, the conclusions remain unchanged if a surrogate variable is used instead. The two WLS approaches are compared to three alternate approaches that use ordinary least squares (OLS). The first OLS approach uses the raw PMR data as the outcome (PMR), the second uses a transformed PMR that is meant to remove the skew (ln(PMR+1)), and the third approach filters the data and only analyzes PMRs from samples that exceed some minimum number of genome equivalents (PMR[*h *> threshold]). We consider thresholds of 1 and 10 genome equivalents. The number 10 genomes is chosen by inspection of Figure 2C, recognizing that the variance in the outcome measure begins to increase dramatically when 10 or fewer genomes are sampled. We also consider 1 genome equivalent as an intermediate value between a threshold of 10 and not using any threshold at all. The thresholding approach is also an implementation of weighted regression, with weights of 1 or 0 depending on whether the DNA quantity is above or below the defined threshold.

## Results

Figure 2 shows the pronounced effect of the DNA quantity on the variation in PMR measures for proportions of methylated alleles of 0.2 and 0.8. In Figures 2A and 2B, the proportion of methylated alleles is the same for all tumors sampled. All of the variation in PMR is due to sampling DNA fragments. In Figure 2C, the proportion of methylated alleles varies among individual tumors, reflecting the variation that occurs in human populations. This variation is modelled using a Beta distribution with mean of 0.2 and variance of 0.01, a uni-modal distribution skewed to the right. The right-most column in Figures 2A-C show boxplots of the PMR values by decile of DNA quantity. The grey dots give the estimate of the mean PMR for each decile, and show that there is no bias in estimating the average PMR as a function of DNA quantity. The decreasing size of the boxes shows the decreased variance of the PMR value with increasing DNA quantity. If the reference sample is not fully methylated, a bias is introduced and the PMR values are overestimated and the variances inflated (results not shown).

**Figure 3:**
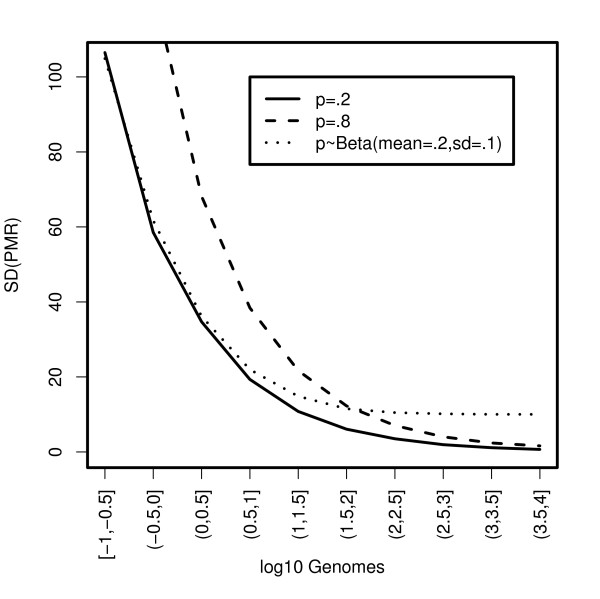
**Plot of Standard Deviation of simulated PMR by decile of input DNA quantity**. p = true methylation proportion.

Figure 3 shows the standard deviation of the PMR values estimated by decile of DNA quantity for the three scenarios in Figure 2. Here we see that the standard deviation is not only a function of DNA quantity, but also of the proportion of methylated alleles. The higher the true proportion of methylated alleles, the greater the variation in PMR values for any quantity of DNA. This relationship is a consequence of the Binomial variance for the number of methylated molecules in the numerator of the PMR formula and that we are analyzing fragmented DNA. Locus-specific methylation frequencies of 0.2 and 0.8 in genomes correspond to frequencies of 2e-5 and 8e-5 in fragmented genomes (= p/10^4^). Interestingly, if we compare the variation for the two models with mean methylation proportions of 0.2, we see that for tumors with large quantities of DNA (e.g. >100 genomes), the standard deviation curve becomes flat when there is population-level variation in the proportion of methylated alleles. This suggests that the population-level variation overwhelms the variation at the tumor level due to sampling DNA fragments. However, for tumors with very little DNA (e.g. <3 genomes), the curves are approximately the same, suggesting that the sampling variation at the tumor-level overwhelms the variation at the population-level.

**Figure 4:**
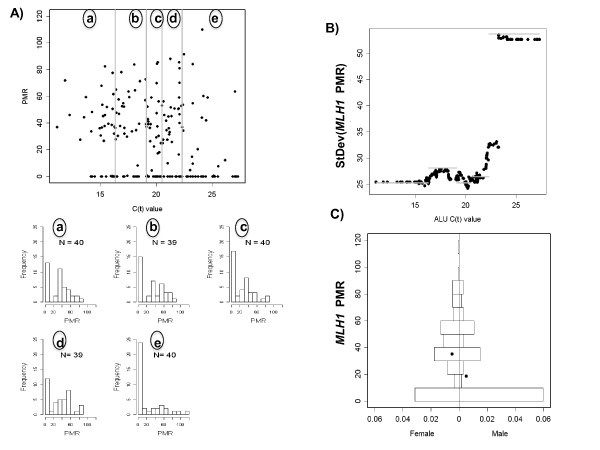
**DNA methylation of *MLH1 *in the Colon-CFR**. A) Scatterplot of *MLH1 *Percent Methylated Reference (PMR) against *ALU *C(t) value for 198 MSI-H colon cancer samples from Seattle. (a)-(e) Histograms of the PMR values by 5 quintiles of *ALU *C(t). B) a plot of SD of *MLH1 *PMR by quintile of *ALU *C(t) (grey bars) and by sliding windows (black circles). C) Back-to-back histograms of the *MLH1 *PMR in females and males for 198 MSI-H tumors from the Colon CFR (Seattle site). One sample with a PMR = 302 lies outside the range of the figure. Black dots denote the overall mean (including the sample not displayed.)

The Colon CFR data displayed in Figure 4 show similar relationships between PMR and DNA quantity, when using *ALU *C(t) value as a surrogate for DNA quantity. The *ALU *C(t) value, the number of PCR amplification cycles needed to reach a specific PCR amplification threshold of detectability, is inversely associated with quantity of input DNA. High *ALU *C(t) values reflect samples with low quantities of input DNA (many amplification cycles needed) whereas low *ALU *C(t) values reflect samples with large quantities of input DNA (few amplification cycles needed) (see Additional file 1 for details.) The earlier result that PMR variance *decreases *with increasing DNA quantity (Figure 3), and the inverse relationship between DNA quantity and *ALU *C(t) value, suggests that PMR variance *increases *with increasing *ALU *C(t) value. This is the relationship observed in the Colon CFR data (Figure 4B).

We used a simulation study to compare the power and false-positive rates of the different statistical analysis methods. The settings are selected to resemble the distributions we observed in our experimental data. Figure 4A shows the distribution of PMR values for the C-CFR experimental data by quintile of *ALU *C(t) value, our surrogate for DNA quantity. The distribution of PMR values among samples with high DNA quantity (Figure 4A, histogram (a)) appears to come from a mixture distribution with a large fraction of tumors unmethylated, and a mean of about 0.45 among the tumors positive for DNA methylation. Our simulations include both pure Beta distributions to describe the underlying population distribution of methylation proportions, but also Beta-Bernoulli mixtures where a fraction of samples have undetectable DNA methylation.

**Figure 5:**
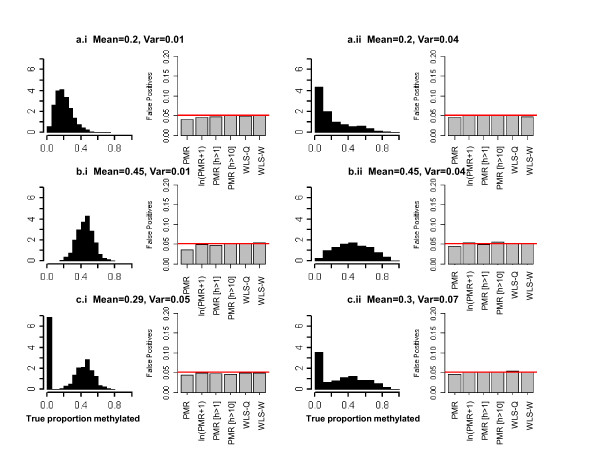
**Histograms showing distributions of true methylation proportions and estimates of false-positive rates (FPR) for six simulation scenarios**. Estimates of FPR from 10,000 replicate data sets of 200 tumors (100 each group).

We tested the different regression approaches under the null hypothesis for six different distributions (Figure 5). Four different distributions are used to capture features related to possible skewness from an underlying Beta distribution in the population. In the last two scenarios (c.i, c.ii), we mimic the Beta-Bernoulli distribution observed in the C-CFR by using the Beta distributions from scenarios b.i and b.ii, with the addition of a Bernoulli variable that independently assigns samples to the positively methylated or unmethylated fractions; the probability a tumor has positive methylation is 67%. We sampled DNA quantities (on the log_10 _scale) using a lognormal distribution. Let *h *be a random deviate from a lognormal distribution with mean of 0 and standard deviation of 0.75 (on the log scale). The log_10 _DNA quantity was equal to 2.74 - *h*. This distribution caps the maximum number of genomes at 550, with 23% of the samples having less than 10 genomes, and 9% having less than 1 genome. The shape of the distribution was chosen to match the shape observed in our epidemiologic study.

Each data set contains 200 tumors, with sample size ratios in two groups of 1:1, 1:2, and 2:1. We ran 10,000 replicates to estimate false-positive rates for a 5% significance level. For sample sizes of 100 in each group, the false-positive rates show that all tests achieve the nominal 5% significance level (Figure 5). Similar findings were observed for sample size ratios of 1:2 or 2:1 (results not shown).

We compared power for nine two-sample distributions with unequal means in the methylation proportion. The distributions of the true methylation proportion are described in Table 1 (see also Additional file 2). The means and variances of the Beta distributions for tumors with detectable *MLH1 *DNA methylation, the Bernoulli probability that a tumor has *MLH1 *DNA methylation, and the differences in overall means and variances between the two groups are provided. Scenarios a and b consider pure Beta distributions, with scenarios for equal variances that are small (scenario i), equal variances that are large (scenario ii), and a larger variance occurring with the larger mean (scenario iii). Scenario c shows a Beta-Bernoulli mixture, where the Beta distribution with higher mean has the greater number of tumors that have positive methylation. This same effect observed here has also been reported by others [1,6].

**Table 1:** The distribution of methylation proportion by patient subgroup (Group 1, Group 2), in the simulation study

	Bernoulli Proportion ^**1**^	Beta distribution ^**2**^			
		Mean	Var		
	
Scenario	(Group 1, Group 2)	(Group 1, Group 2)	(Group 1, Group 2)	Difference in Overall Means	Difference in **Overall Vars**^**3**^
a.i	(1,1)	(0.2, 0.27)	(0.01, 0.01)	0.07	0
a.ii			(0.04, 0.04)	0.07	0
a.iii			(0.01, 0.04)	0.07	0.3
b.i		(0.4, 0.47)	(0.01, 0.01)	0.07	0
b.ii			(0.04, 0.04)	0.07	0
b.iii			(0.01, 0.04)	0.07	0.3
c.i	(0.5,0.7)	(0.4, 0.47)	(0.01, 0.01)	0.13	0.01
c.ii			(0.04, 0.04)	0.13	0.01
c.iii			(0.01, 0.04)	0.13	0.02

^1 ^The probability that the true methylation proportion is positive

^2 ^Distribution of the true methylation proportion in methylated tumors

^3 ^Estimated from the data.

**Figure 6:**
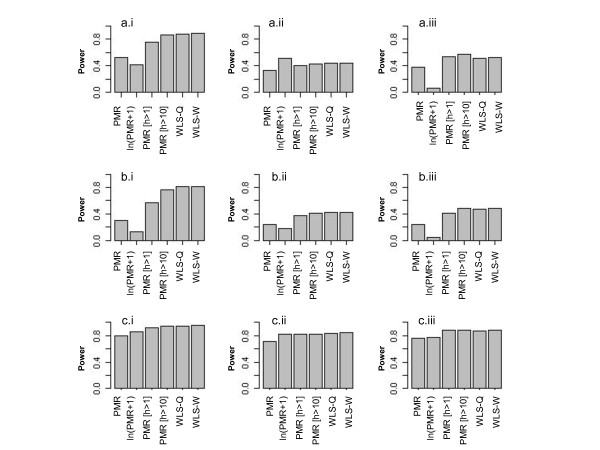
**The simulated power comparing the six analysis methods**: PMR, ln(PMR+1), PMR[h > 1], PMR[h > 10], WLS-Q and WLS-W (1000 replicate data sets, N = 200 tumors total) under nine simulation scenarios.

Figure 6a.i shows the empirical power for the six analytic approaches in our simulations. In scenario a.i, the true DNA methylation proportions are simulated from Beta distributions with means of 0.2 and 0.27 (difference = 0.07) and variances of 0.01. The power to detect differential DNA methylation is 56% if we analyze the raw PMR values using ordinary least squares, and only 41% if using the log-transformed data. However, the power improves to 88% using either weighted least squares approach, WLS-Q or WLS-W. The analysis that omits data based on a threshold for DNA quantity shows intermediate power. The power is 76% after omitting tumors with less than one genome equivalent and 86% after omitting tumors with less than 10 genomes.

For all scenarios, we find the power for the WLS-Q and WLS-W approaches to be very similar (Figure 6). They both outperform the OLS analysis of the raw PMR measure for all instances, and they outperform the analysis of the log-transformed data for all but one example. The most improvement in power occurs when the mode is non-zero. For Beta distributions with low mean and high variance, the distribution appears exponential near zero with skew to the right. For this situation (Additional file 2, box a.ii), OLS using the log-transformation turns out to be slightly more powerful than the WLS approach on the untransformed data (Figure 6, Barplot a.ii). However, further analysis suggests that the best approach for this situation is to perform weighted regression on the log-transformed data. The power for WLS-W on the log-transformed PMR values is 0.62, whereas it was only 0.51 using OLS. Finally, the OLS method that excludes data based on a threshold for DNA quantity often outperforms the other OLS methods. The optimal threshold depends on the distribution of DNA methylation in the population. These results show that it is advantageous to exclude samples with low quantities of DNA with a smaller sample size remaining for statistical analysis than treating them equally to low-variance (high-quality) measures.

### *MLH1 *analysis in Colon CFR Study

We report results for a set of 198 microsatellite instable (MSI-H) tumors from patients recruited at the Fred Hutchinson Cancer Research Center, Seattle, Washington C-CFR site. This subset is selected because very few methylated samples were observed in the other MSI categories and because Seattle is the site that recruited the largest proportion of MSI-H cases among all of the participating C-CFR sites. This sample set showed a median *ALU *C(t) of 19.8 with an interquartile range 16.9 to 22.0. Applying the results from the supplementary study (see Additional file 1), we estimate that half of the tumors provided at least 46 genomes for molecular analysis (interquartile range: 15-204 genomes), 25% provided less than 15 genomes and 10% less than six genomes.

Poynter et al. (2008) found a number of variables associated with *MLH1 *DNA methylation in MSI-H tumors. Here, we performed the five analyses above to study the association between *MLH1 *DNA methylation and sex. Figure 4C shows back-to-back histograms of the PMR values in males and females. In their analysis, Poynter et al. (2008) excluded samples with an *ALU *C(t) above 24. The threshold was selected empirically by trying to maximize sample size while maintaining sensitivity to detect DNA methylation. Applying the three OLS methods PMR, ln(PMR+1), PMR[ct < 24], we estimated p-values of: 0.001, 5.4 × 10^-5^, 0.001, respectively. Because of the mode occurring at zero, it is not surprising that the analysis of the log-transformed data provides the most statistically significant result. Using WLS on the raw PMR measures we estimate p-values of 3.2 × 10^-4^, and 2.5 × 10^-4^, for the WLS-Q and WLS-W approaches, respectively. The p-values became more statistically significant when performed on the log-transformed data, 5.5 × 10^-5^, and 4.3 × 10^-5^. This suggests that for data with a mode of zero, the WLS approach is more sensitive when conducted on the log-transformed data.

## Discussion

As molecular technologies have evolved and costs have dropped, the evaluation of tumor heterogeneity is now feasible in large population-based epidemiology studies. In such studies, having small amounts of input DNA in a subset of the samples has the potential to obscure results if not properly analyzed. Poynter et al. (2008) recognized this issue. Viewing *ALU *C(t) as a surrogate measure of DNA quantity, they eliminated samples with high *ALU *C(t), or equivalently low quantity input DNA, from their statistical analysis. Their approach is equivalent to a weighted regression analysis, with weights of 1 for measures from tumors with DNA quantity above a fixed threshold and 0 otherwise. We propose a more flexible WLS regression approach to incorporate variation due to sampling from small quantities of tumor DNA. The approach uses empirically derived weights by using the inverse variance of PMR as a function of a surrogate measure of DNA quantity. There may be more than one approach to quantify DNA for this purpose. For example, A_260_nm absorbance measurements may also be used. The method requires stable estimates of variance obtained from large sample sizes, making it both feasible and practical in large epidemiology studies.

Several gene expression microarray studies have also used weighted regression to account for variable data quality [7-9]. They use the large number of genes measured on a single array to capture information on quality, and do not limit the quality to only one aspect such as DNA quantity. In our application only one gene region is measured, and quality is estimated by the variance in the measure as a function of quantity of input DNA analyzed.

In a simulation study, we find that a WLS approach has better power to detect differential DNA methylation between groups of patient samples than standard methods that ignore the variation due to measuring small quantities of DNA. When the data have a mode at zero with skew to the right, the most powerful approach is to perform a WLS regression on the log-transformed data. Slightly less powerful than the WLS-Q and WLS-W approaches is the OLS approach that simply excludes samples with low DNA quantity. As described earlier, this is also a WLS approach, with weights of 0 and 1, and by itself always outperforms OLS on the raw PMR values. We show that it can be possible to select a good threshold based on plotting the variation in the outcome measure as a function of a surrogate measure for DNA quantity. Nevertheless, a more quantitative model for the weights can show higher power than this dichotomous weighting scheme.

The difference in power between OLS and WLS approaches will depend on the distribution of DNA quantity and its association with other study variables. Throughout all our analyses, the DNA quantity distribution followed a log-normal distribution (on the log_10 _scale) with 9% of the samples having less than one genome equivalent of input DNA. This distribution can be expected to vary from study to study. If all tumors have large quantities of DNA, the variation due to sampling is negligible compared to the population-level variation in methylation proportions; in this case, the unweighted and weighted regressions will perform similarly. For the level of population-variation used in our simulations, the variation due to sampling DNA overwhelms the population-level variation somewhere between 1 and 10 genomes. In our simulation study, even a naïve weighted regression approach that omits 20% of the data with low DNA quantities is more powerful than the standard regression analysis, despite the reduction in sample size. However, when group variances are homogeneous, it appears that an approach that estimates weights in a continuous range between 0 and 1 is preferred to one that simply excludes samples using a threshold.

Overall, we saw little difference between the WLS-Q and WLS-W methods. This is presumably a function of sample size, and larger sample sizes may allow us to see an improvement when using local windows. Our simulation study was performed using a sample size based on the Colon CFR study (N = 200), however the approaches might also be applicable for smaller sample sizes. The stability will depend on how reliably the variances can be estimated from the data, and for this we used a window size of 40 observations. This seemed to be the minimum window size that would allow us to control the type I error. An analysis of 100 observations and window size of 20 resulted in an inflated type I error, however a sample size of 120 observations and window size of 40 did not. The potential to identify differences between the two weighted regression approaches (WLS-Q/WLS-W) will also depend on the empirical association between variance of our outcome measure and DNA quantity. The smoothing method is likely only to show greater power if the variation in outcome measure changes across a range of DNA quantities in a way that we cannot capture by a discrete distribution. For our Colon CFR data set, it appears that the discrete distribution is sufficient.

The weighted regression approach assumes that the variation in PMR due to low DNA input is independent of other study variables. For the multi-center C-CFR study, the *ALU *C(t) distribution, our surrogate measure for DNA quantity, was independent of patient-level variables such as sex and age of disease onset, but varied between C-CFR centers. Since the sampling design also varied by center, this could complicate the analysis in ways that are beyond the scope of this report.

Finally, despite the fact that our experimental data appeared to be from a continuous distribution that included an excess of values at zero, we chose to ignore the discrete nature of the data at zero and only evaluate regression models for a continuous variable. One could propose the use of a two-degree of freedom test to simultaneously examine the difference in proportions of tumors with detectable DNA methylation, as well as the difference in means levels for those tumors. However, previous work has found that when the differences in means and proportions occur in the same directions, as is the situation for our data, the one degree of freedom test from an analysis of the quantitative variable is more powerful [10]. This improvement occurs because the difference in the overall mean for the two groups is larger than the difference among the positive tumors only with fewer degrees of freedom tested. The only instance in which a two degree of freedom test is more powerful than the one degree of freedom test is when the difference in fractions of tumors having DNA methylation and the difference of the means for those fractions, occur in opposite directions. The latter situation is not observed in our data, supporting our choice for using regression models for continuous outcomes.

An alternate approach, and one often taken for DNA methylation data, is to dichotomize the quantitative measures into "methylated" and "non-methylated" fractions. However, when a subset of tumors contains low quantities of DNA, the ability to detect DNA methylation at a target site within a tumor decreases, resulting in false-negative outcomes. In this situation, the mean methylation fraction is no longer unbiased as DNA quantity decreases, and instead underestimates of the methylation fraction are obtained. Thus for dichotomous data, our approach does not provide unbiased estimates of the fraction of tumors methylated and further research on how to weight the analysis for this situation is still needed.

## Conclusions

In molecular analysis of tumor tissue, the variation in features measured is a combination of the population-level (subject-to-subject) variation in tumor characteristic as well as the individual-level (within-tumor) variation due to sampling genomic DNA. When large amounts of genomic DNA are used for molecular analysis, the features are well measured and the variation in measures reflects population-level variation. However, as the amount of input DNA decreases, the molecular features under study are measured with error. They can be missed (false-negatives) or appear as outliers beyond the normal range of the data. A careful statistical analysis will want to consider the sampling variation introduced at the tumor-level due to low quantities of input DNA.

## Competing interests

CR, DJW, PM and KDS declare that they have no competing interest. PWL is a consultant and scientific advisory board member for Epigenomics, AG, which has a commercial interest in DNA methylation markers. Epigenomics did not fund any part of this study.

## Authors' contributions

The manuscript was written by CR, DJW, and KDS KDS conceived of the WLS approaches and performed the statistical analyses, DJW performed the analysis of *ALU *C(t) values and DNA quantity, PM served as an advisor, and PWL conceived the project. All authors read and approved the final manuscript.

## Supplementary Material

Additional file 1 **Inverse relationship between *ALU *C(t) value and DNA quantity **Detailed description of the additional experiment from which we estimated DNA quantity from *ALU *C(t) value. Click here for file

Additional file 2 **Back-to-back histograms of true methylation proportions under 9 alternative hypotheses**. Figure displaying the distribution of the simulated data under the 9 chosen alternative hypotheses. Click here for file
