# Supplementary material for: Modeling measurement error in tumor characterization studies
Source: BMC Bioinformatics. 2011 Jul 13;12:284. doi: 10.1186/1471-2105-12-284 (PMC3213130; doi:10.1186/1471-2105-12-284)
Supplement: Additional file 1 — Inverse relationship between ALU C(t) value and DNA quantity Detailed description of the additional experiment from which we estimated DNA quantity from ALU C(t) value. [file 1471-2105-12-284-S1.PDF]

Additional file 1 – C(t) value and DNA quantity

The formula below describes the inverse relationship between *ALU* C(t) value and input DNA. Let τ and c be amplification process-specific constants denoting the DNA quantity detection threshold and the method's efficiency. The efficiency is a composite of the amplification efficiency and background levels. Further, let h denote DNA quantity (in genome equivalents), and C(t) denotes the corresponding number of amplification cycles needed to enlarge h to a DNA quantity exceeding τ. That is,

$$\mathrm{C(t)} = \min\{i \geq 1\colon hc^{i} \geq \tau\}. \qquad (1)$$

The approximate formula for the relation between C(t) and DNA quantities is obtained from (1) after replacing the inequality with equality,

$$hc^{C(t)} = \tau. \qquad (2)$$

Taking logarithm with base 10 on both sides of (2) and rearranging terms yields,

$$\mathrm{C(t)} = (\log_{10}\tau - \log_{10} h)/\log_{10}\mathrm{c}$$

$$= \alpha - \beta \log_{10} h \qquad (3)$$

This formula shows the inverse relationship between DNA quantity and C(t) value, allowing us to use *ALU* C(t) value as a surrogate for DNA quantity in our study.

We designed a supplementary experiment that would allow us to estimate the quantity of DNA for each tumor analyzed given the surrogate *ALU* C(t) value. This is accomplished through the use of a TaqMan PCR reaction (C-LESS-C1)[11], which recognizes a DNA strand that does not contain cytosines, and hence is able to amplify the total amount of DNA (bisulfite-converted or unconverted) in a PCR reaction well. The supplementary experiment is based on the analysis of two serially diluted sets of DNA samples: (1) an *unconverted* peripheral blood leukocyte (PBL) DNA sample of known concentration for use as a standard curve in a C-LESS PCR reaction, and (2) a

bisulfite-converted M.*Sss*I-DNA sample used in *ALU* PCR reactions of the C-CFR *MLH1* DNA methylation analyses. In order to convert the C-LESS PCR standard curve from the PBL sample into an *ALU* standard curve for the C-CFR *MLH1* samples, the dilution series of bisulfite-converted M.*Sss*I-DNA sample (set 2 above) was analyzed using both C-LESS and *ALU* MethyLight PCR reactions.

First, we used the serially diluted set of unconverted PBL DNA sample of known concentration to estimate the C-LESS C(t) value as a function of the known concentration (463.2 ng DNA undiluted, as measured by Nanodrop-based A_{260} absorbance technology) and relative number of copies (1024, 256, 64, 16, 4, and 1 for dilutions: undiluted, 1:4, 1:16, 1:64, 1:256, 1:1024). We re-wrote the best-fit linear equation to predict ng of unconverted DNA (on the $\log_{10}$ scale) as a function of C-LESS C(t) value. Since the C-LESS reaction amplifies both template strands of unconverted genomic DNA but only one strand of bisulfite-converted DNA, we expect that bisulfite-converted DNA will amplify one PCR cycle later than that of unconverted DNA. Thus, to estimate the number of haploid bisulfite-converted DNA for the M.*Sss*I-treated DNA sample, we multiply the amount of DNA (in ng) for each sample quantitated from the C-LESS standard curve by the PCR efficiency, and divide by 0.0033 (1 haploid genome = 0.0033 ng DNA). We calculated the estimated C-LESS PCR efficiency in this experiment to be 1.905. Next, we generated a regression of the *ALU* C(t) value on the estimated number of haploid genomes (on the $\log_{10}$ scale) for the dilution series for the M.*Sss*I-treated DNA sample (dilutions of 1:729, 1:243, 1:81, 1:27, 1:9, 1:3, 1:1). The final step was to calibrate the dilution set from the 28 C-CFR plates to the same reaction run coincidently with C-LESS. We estimated a regression function of the *ALU* C(t) level from the reaction run coincidently with the C-LESS reaction as a function of the average *ALU* C(t) level

estimated across the 28 C-CFR plates. Thus, by knowing the *ALU* C(t) level of a tumor in the C-CFR samples, we could then estimate the number of haploid genomes.
