# Supplementary material for: Modeling measurement error in tumor characterization studies
Source: BMC Bioinformatics. 2011 Jul 13;12:284. doi: 10.1186/1471-2105-12-284 (PMC3213130; doi:10.1186/1471-2105-12-284)
Supplement: Additional file 2 — Back-to-back histograms of true methylation proportions under 9 alternative hypotheses. Figure displaying the distribution of the simulated data under the 9 chosen alternative hypotheses. [file 1471-2105-12-284-S2.PDF]

Additional file 2:

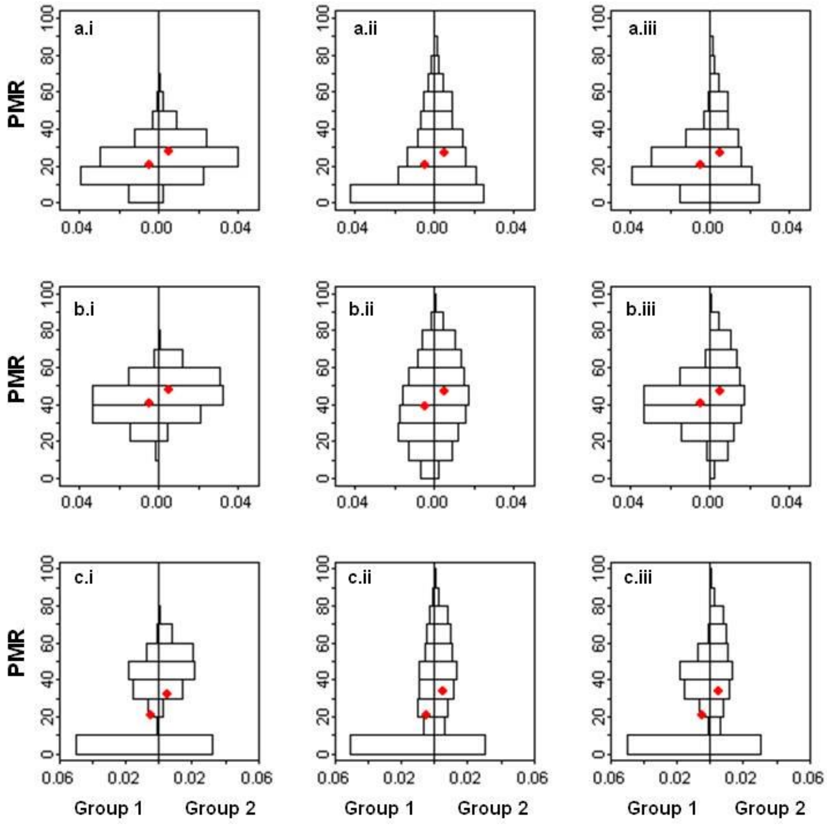

Supplementary Fig. 1. Back-to-back histograms of true methylation proportions under 9 alternative hypotheses. Colored dots denote the overall mean PMR in the group.
